# Internet of Things for Smart Spaces: A University Campus Case Study

**DOI:** 10.3390/s20133716

**Published:** 2020-07-02

**Authors:** Ekaterina Gilman, Satu Tamminen, Rumana Yasmin, Eemeli Ristimella, Ella Peltonen, Markus Harju, Lauri Lovén, Jukka Riekki, Susanna Pirttikangas

**Affiliations:** 1 Center for Ubiquitous Computing, University of Oulu, P.O. Box 4500, 90014 Oulu, Finland; ella.peltonen@oulu.fi (E.P.); lauri.loven@oulu.fi (L.L.); jukka.riekki@oulu.fi (J.R.); susanna.pirttikangas@oulu.fi (S.P.); 2 Biomimetics and Intelligent Systems Group, University of Oulu, P.O. Box 4500, 90014 Oulu, Finland; satu.tamminen@oulu.fi (S.T.); markus.harju@oulu.fi (M.H.); 3 Centre for Wireless Communications, University of Oulu, P.O. Box 4500, 90014 Oulu, Finland; rumana.yasmin@oulu.fi (R.Y.); eemeli.ristimella@student.oulu.fi (E.R.)

**Keywords:** ubiquitous computing, ambient intelligence, data analysis, Internet of Things, smart space, smart campus

## Abstract

Advances in technology and data analysis provide rich opportunities for developing intelligent environments assisting their inhabitants, so-called smart environments or smart spaces. Enhanced with technology, sensors, user interfaces, and various applications, such smart spaces are capable of recognizing users and situations they are in, react accordingly, e.g., by providing certain services or changes to the environment itself. Therefore, smart space solutions are gradually coming to different application domains, each with corresponding specific characteristics. In this article, we discuss our experiences and explore the challenges of a long-term real-world Internet of Things (IoT) deployment at a University campus. We demonstrate the technical implementation and data quality issues. We conduct several studies, from data analysis to interaction with space, utilizing the developed infrastructure, and we also share our actions to open the data for education purposes and discuss their outcomes. With this article, we aim to share our experience and provide real-world lessons learned when building an open, multipurpose, publicly used smart space at a University campus.

## 1. Introduction

Expansion of Internet of Things (IoT) solutions, cloud, mobile, and edge computing, as well as advances in data analysis, provide now rich opportunities for developing intelligent environments able to assist their inhabitants, so called smart environments or smart spaces [1]. Such environments are equipped with technology, sensors, user interfaces, and applications that are capable of recognizing users and situations they are currently in. Together with development of smart spaces, the idea of supporting learning and teaching with technology has emerged [2]. Therefore, smart spaces research also comes to university campuses, including, but not limited to, measuring well-being of the students and teachers, tracking learning outcomes, and providing varying technologies for novel pedagogy needs. In this article, we share our experiences from a real-world IoT deployment to multipurpose open space shared by many visitors at a University campus. Our IoT installation in Tellus Innovation Arena premises at the University of Oulu is the first step towards the University of Oulu smart campus.

Smart spaces identify the user needs and react accordingly, for example, by providing the corresponding services or changes to the environment itself. In addition to personalized applications, smart spaces enable us to obtain insights into space usage and our interactions within and with the space. This can help, for example, to rearrange the furniture to serve the users better in a timely manner or adjust the temperature and air conditioning of the space. Data that are required for space usage analysis and optimization come from sensors and devices of the smart space and gadgets that are carried by the visitors. Statistical and machine learning methods help to understand the situation from the collected data and infer which actions to perform in the smart space.

Smart environments in open and public spaces—which university campuses are—pose a number of challenges. First, open spaces often host a variety of activities, i.e., they are multipurpose. Therefore, targeting one particular application domain could be unfeasible. Second, public spaces are shared by multiple visitors at the same time. Thus, tailoring space to a specific participant is tricky. Third, some smart space applications rely on the use of personal devices. Personal devices, as well as the devices of smart spaces, have varying capabilities, requiring adaptive execution environments for the smart space applications [3]. Fourth, less attention is given to make people aware of the sensing and interactive capabilities of smart spaces. For instance, how to indicate that object possesses interactive abilities not disturbing its original outlook and functionality [4] or how to deliver IoT information in an understandable and convenient manner to a visitor [5]. Fifth, transparency and traceability of decisions made by applications in such multi-user smart spaces become even more challenging [6]. Finally, to obtain meaningful results, special care should be taken when deciding the number, type, and location of sensors to deploy, as the quality of information may suffer from poorly placed or selected sensors. To summarize, challenges in public smart space deployments vary from reception of the public to technical difficulties in implementation, quality of measurements, and data analysis. In this article, we highlight the aspects from data quality and analysis to communication and interaction with the users, where from implementation issues also straightforwardly emerge.

University campuses provide vibrant opportunities for testing diverse smart solutions, as these environments are complex ecosystems, involving different stakeholders, sectors, processes, and systems. Moreover, University campuses are usually more open for such experiments, as being the centers for professional education and research they have the required multidisciplinary expertise and scientific and development knowledge. In the literature, researchers even refer to campuses as small-scale cities [7,8]. Indeed, some consider that certain aspects can first be studied within campuses and scaled up to smart city use cases, like smart transport, smart building, and smart parking [9].

Tellus Innovation Arena (Tellus) at the University of Oulu, Linnanmaa campus, is an open space that is designed for collaborative work, study, presentations, and includes various kinds of spaces, like meeting rooms, open stage area, cafeteria, study, and rest areas. By equipping this place with sensor technology, we aim to understand the space usage better, explore what kind of applications would suit such space and would be beneficial for visitors, as well as highlight challenges observed and lessons learned. Smart campuses and similar technology-oriented environments have become trendy wish everywhere in the world. Still, there is a lack of publicly shared critical experiences and lessons learned when building such environments for actively used multipurpose spaces. Thus, sharing experiences and challenges of real-world implementations becomes crucial for further smart campus developments—the gap that we are filling with this article.

To summarize, the contribution of this article is as follows:

1. We present a longitudinal IoT deployment in the shared open space within the University campus. We discuss the lessons we have learned based on our experience in equipping public open space with IoT technology at the University campus.
2. We present a number of studies exploring how this shared open space is actually used, how its state can be communicated, and how users can interact with this space.
3. We share experiences in using this IoT deployment in teaching and development activities. We discuss challenges and possibilities to bring the users of the space along with its further development.

This article is structured, as follows: Section 2 reviews related work with the focus on the challenges of shared spaces. Section 3 introduces our real-world IoT deployment for Tellus that assists students and university staff in various kinds of collaborative work. In Section 4, we go through a number of studies, insights, and experiments conducted at Tellus. Section 5 discusses the challenges and lessons learned from deployment of IoT solution in open shared space at a University campus. Finally, our article is closed with Section 6.

## 2. Related Work

Essentially, to support their inhabitants, smart spaces need to be context-aware: detect and react to context (i.e., information characterizing situation in smart space) changes [10]. In addition, technologies equipping smart space can “weave themselves into the fabric of everyday life until they are indistinguishable from it” [11]. Ubiquitous computing, pervasive computing, and ambient intelligence research fields align with these notions of context-awareness and disappearing computing. However, many research articles use these terms interchangeably. In this article, we refer to an Ambient Intelligence (AmI) system as “a digital environment that proactively, but sensibly, supports people in their daily lives” [12]. Relying on their multidisciplinary nature [13], in addition to context-awareness, disappearing computers, ubiquitous and pervasive computing, AmI systems emphasize the necessity of “intelligence” as a fundamental element by relying on advances in artificial intelligence, machine learning, agent-based software, and robotics [14]. Hence, in this article, to put a focus on “intelligence”, under smart space we essentially consider an AmI system.

A considerable body of research exists regarding smart spaces and their prototyping in different application domains [13]. For example, research on the implementation of AmI systems explores system architectures and middleware [15]. Second, context modeling research looks at solutions for interoperability, while context reasoning and decision-making research focus on insights leading to actions [16]. Third, Human-Computer Interaction (HCI) and social acceptance of AmI systems explore how users interact with such systems [17]. Finally, research on ethics dives deeper to the ethical and legal aspects of AmI systems [18]. Further, the number of prototypes focusing on personalization is provided in different application domains, like smart homes, smart offices, or smart healthcare, to name but a few [13,19].

Our work focuses on a real-world IoT deployment for a multipurpose shared open space at the University campus. Since we consider such deployment as a first step towards smart campus, the literature review presented here will focus on two main aspects: (1) shared smart spaces and corresponding challenges and (2) smart campus solutions that are available in related work.

### 2.1. Smart Shared Spaces

Libraries, public squares, open work places, and meeting places are examples of shared spaces. For instance, ambient intelligence technologies provide excellent opportunities for shared learning environments, as they can enable adaptation of learning applications and activities to student learning needs and progress [2,20,21]. These spaces, like classrooms or laboratories, although multi-user, are often controlled by teachers, who actually design learning activities and create the content. In addition, students often possess their own devices through which personalized learning services and feedback are delivered, e.g., mobile phones or tablets [22]. Another interesting application domain of shared spaces includes hospitals and health care units. These spaces are characterized by highly distributed nature of information available, from personal sensors to medical records. However, the majority of smart systems in healthcare are targeted for medical personnel only, with strong authentication mechanisms to connect to available infrastructure and devices due to the sensitivity of health-related data [13,19]. Some scenarios for shared spaces also include patients, like that by Rodriguez et al. [23], where a patient gets informed by the smart system if one of the patients of the doctor, recommended to her by the system, is in the same waiting room, in case she wants to approach her for discussion.

Equipping spaces shared by multiple users with technology to provide personalized utility services is challenging. That is why many such systems use personal devices to sense the space activity and to deliver utility services [22,24]. Active use of multiple shared resources in smart space (e.g., public screens) seems to be less common [25]. Here, we purposefully omit various architectural solutions for smart space implementation already discussed in literature [16,26,27]. Instead, we concentrate on data quality issues related to decision-making in smart spaces, challenges for interaction in shared smart space, and ethical concerns.

**From data to actions**. Smart space applications sense, interpret, and act according to the situation. Hence, identifying the situation is necessary to choose the action to perform. Here, the situation can be defined as “external semantic interpretation of sensor data”, where interpretation means that meaning is assigned to the sensor data, and external means that interpretation is given from the applications’ perspective [28]. Therefore, the identification of the situation occurs from a variety of data sources, like sensors, video data, personal information, etc. These data aggregate into a set of domain concepts or context [10,28]. Subsequently, more complex context structures can be created from an elementary context, finally leading to situation abstraction [28]. For example, from sensor readings, an activity, like “eating” or “sleeping”, could be inferred, that could lead to further smart space application actions to help the user to achieve the task [29]. Plenty of research has been conducted on different aspects that are related to context acquisition, modelling, and reasoning in smart spaces [16,26,27]. Here, we would like to highlight some aspects related to data quality and analysis in smart spaces.

Since, in smart spaces, the situation is identified from data, it is essential that the data provided by sensors are of sufficient quality, as data fault may further propagate to the misbehavior of the system [30]. Sensor data can be voluminous, erroneous, and noisy. Therefore, appropriate solutions should be in place to deal with unknown, ambiguous, imprecise, and erroneous data [31]. For example, sensor specifications alignment to smart space and setup is essential. Some design or operational characteristics may not suit a particular smart space. Additionally, sensors should be positioned in such a way that the phenomenon of interest is captured fairly, e.g., temperature sensor measuring room temperature should not be placed near any heating object. The same issue applies to wearables and personal devices used for crowdsourcing data [32]. Another issue is the possible interference of devices affecting the quality of data.

Additionally, activity recognition itself becomes much trickier in a multi-user environment [28,33]. To approach this issue, different temporal and behavioral aspects of users can be studied, e.g., time of the day, day of the week of certain activity [34]. Another solution that is based on multi-label classification is proposed [35], allowing for detecting several activities of multiple users. Technological infrastructure of the space dictates if multi-user activity detection is possible, e.g., not all spaces allow installing cameras or wearables may not be available.

We would like to also note that smart spaces are non-stationary environments, where, for example, user preferences may change over time for a variety of reasons or some context information may exhibit rapid change [36]. In a non-stationary environment, the data distribution may change over time; this phenomenon is called a concept drift [37]. In such circumstances, the system should be able to adapt to a changing situation to keep providing adequate support to the users. Here, one of the challenges is to distinguish a real change in the data from a sudden deviation or anomaly, known as an outlier, and approaches have been suggested to determine when adaptation is necessary [36,37].

**Interaction experience**. Smart space applications are often dynamically composed of software components and resources of the space to address the needs of the user [38]. To make interaction experience with such a system comfortable, shared smart space applications should be designed to support users in such a way that other users are also taken into account [39]. This often means that such systems should be able to handle possible conflict cases, e.g., when smart space applications use shared resources. Several approaches are suggested by the research community. For instance, sort of “rules of engagement” can be hard-coded into the system, describing the interaction scenarios, e.g., based on context, personal profiles, service priorities, and technical requirements [40]. Another approach to handle resources is lease-based resource management [41]. There, a lease is a “negotiated agreement between the mobile client and the resource management infrastructure of the smart space, regarding the resources the user needs in performing her task” [41]. Hence, lease facilitates validation of context, technical requirements, spatial and temporal criteria, and allows freeing the resource when criteria are not met anymore. User roles could be considered for conflict management [41]. Some systems can also deliver a “compromised” alternative, or they could explicitly confirm the action from the user [5]. The adaptation or utilization of shared resources in smart space presents a number of technical issues [25,38]; however, it also touches user concerns and feelings about exposing own preferences or affecting others [25,39,42]. Therefore, more recent research considers multi-user interaction as a more challenging concept and explores the possibilities of social translucence, that is, systems should not try to solve all of the conflicts by themselves, instead “interfaces should make socially salient information visible, leading to awareness and accountability in interaction” [39,43].

**What if I do not want to use smart space functionality?** Smart space solutions rely on data that come from infrastructure, third-party services, and users. Therefore, smart spaces must assure that user privacy is respected, and no sensitive and personal information is retrieved and inferred from the person without his/her explicit permission to do so. Large shared spaces are challenging to retrieve user permissions to collect and use the data, especially because not every visitor of space is willing to install a particular application to her mobile phone. On a larger scale, e.g., in the case of the smart cities, it is a challenge even to inform citizens about all of the data that could be collected [44].

Therefore, there is a strong demand to embed not only privacy and security mechanisms, but also ethical data management and processing, as a formal part of system development [6]. For instance, the ethical design is put forward in IoT [45] and intelligent systems for healthcare [46]. A number of initiatives address the ethical challenges of intelligent systems. For instance, the IEEE Global Initiative on Ethics of Autonomous and Intelligent Systems provides principles and practical recommendations for intelligent systems design and development [47]. High-Level Expert Group on Artificial Intelligence (AI HLEG), an independent expert group set up by EU, recently released The Ethics Guidelines for Trustworthy Artificial Intelligence, which sets out a framework for achieving trustworthy AI with the focus on ethics and robustness [48]. Such efforts promote implementation-oriented guidelines to embed ethics in advanced intelligent systems in entrepreneurial global context [6], making a considerable shift towards the development of technologies aligned with values defined by society.

### 2.2. Smart Campuses

Smart campus concept becomes more and more ubiquitous today [49]. Many definitions exist on what “smart campus” actually is [49,50]. Muhamad et al. [49] categorize smart campus definitions into three categories: technology-driven, smart city concept adoption, and organization or business process driven. In the technology-driven definitions category, smart campuses evolve from the digital ones focusing on advanced infrastructure, like sensors and systems, providing diverse services to the users. The adoption of smart city concept puts the focus on the similarities between cities and campuses, supporting various users to perform multiple tasks in multi-functional buildings [49]. Finally, organization or process driven definitions emphasize optimization criteria, e.g., implementing intelligence through the use of various sensor technologies automatically supporting reporting into all aspects of campus life, including learning, social interaction, and intelligent building management [49]. Galego et al. [51] emphasize the need for the human dimension in defining the meaning for “smartness”, most commonly used with more infrastructural focus. The researchers also refer to smart campuses as the environments where different ubiquitous learning systems have been deployed at the campus [2,52].

A number of initiatives exist towards implementing smart campuses [53]. Experiences are shared in integrating and analysis of available data, like Lancaster University integrates data from existing energy and building management systems for analysis and optimization [8]. Authors emphasize opportunities, as well as challenges for such solutions, e.g., related to data capture, analysis, and consequent decision-making. Additional devices are also installed, like in Instituto Superior Técnico in Lisbon [54], where the focus is on energy efficiency and ICT equipment was installed in the library, lecture hall, and some offices, and a number of applications were developed to express preferences and control the environment, like lighting. Wang et al. [55] also implemented an IoT framework for energy optimisation; their first smart campus prototypes, equipped with SVM machine learning algorithms, are able to distinguish between regular and irregular events in the classroom. Webb and Hume [56] share security and governance experience with large IoT deployment at West Texas A&M University. Deployments also include equipping campuses with sensor technologies and delivering sensor information to the users through e.g., interactive Web sites, like Prandi et al. [57].

The majority of such systems rely on IoT and cloud solutions. For instance, Guo and Zhang [58] present technical details of the IoT and Data cloud infrastructure for smart campus in the Wuhan University of Technology. They point out the importance of the cloud stack for data interoperability, storage, and analysis. Haghi et al. [59] utilize Microsoft Azure cloud services to develop smart campus IoT solutions. Recently, several examples exist towards utilizing edge computing in smart campus platforms and applications [60,61]. Such proposals promise minimal delays and, therefore, better quality of services provided.

This article shares the experience from the real-world IoT implementation to the University of Oulu’s Linnanmaa campus, similar to related efforts, e.g., [55,58]. Infrastructure, data analysis, and the number of studies, discovering how space is used and can be interacted with the users, are presented here to highlight numerous opportunities for bringing smart space solutions to campuses. Some studies presented here somewhat intersect with related work, e.g., events detection [56] or visualizing the sensor information through interactive Web pages [57]. Others explore more novel ideas in the context of university campuses, like delivering the state of the environment with augmented reality and environment control in the shared space. Since the space we are dealing with in this article is multipurpose and open for visitors for various activities, we bring attention to the challenges of smart shared spaces, both theoretically (e.g., refer to Section 2.1) and with real implementation (e.g., refer to Section 4.4). Additionally, different from related efforts, we experiment with opening the infrastructure and data for the educational purposes, and discuss what was achieved from this experience. Finally, despite the fact that the topic of smart campuses emerges, we find that there is a lack of sharing the lessons and challenges, especially in shared multipurpose environments [53]. Therefore, together with very few related works, e.g., [8], this article contributes to the effort in bringing in critical issues to be considered when designing and implementing smart campus solutions.

The University of Oulu has started the Smart Campus initiative with the goal to integrate available connectivity, IoT, and computational solutions for more open and efficient use for both research and education. Tellus IoT deployment [62,63], use case of this article, is one of the first steps towards this initiative, and the lessons learned will help us on our pathway towards smart campus.

## 3. Tellus: Towards a Smart Space

Tellus Innovation Arena (Tellus) is an open space in the University of Oulu designed for collaborative work, study, and presentations. We have equipped Tellus with an IoT sensor network, analyzed the data that were collected by the sensors, developed visualization for the data, and utilized this setup in teaching. This section presents infrastructure details and insights on Tellus data.

### 3.1. Tellus Infrastructure

**Figure 1:**
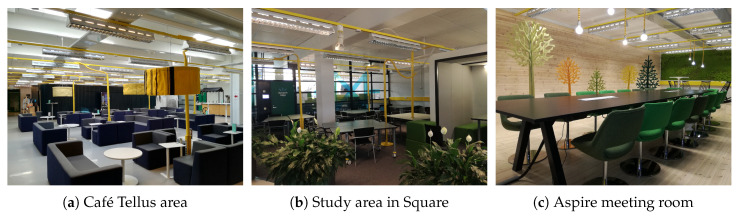
Tellus Innovation Arena (Tellus) spaces.

**Figure 2:**
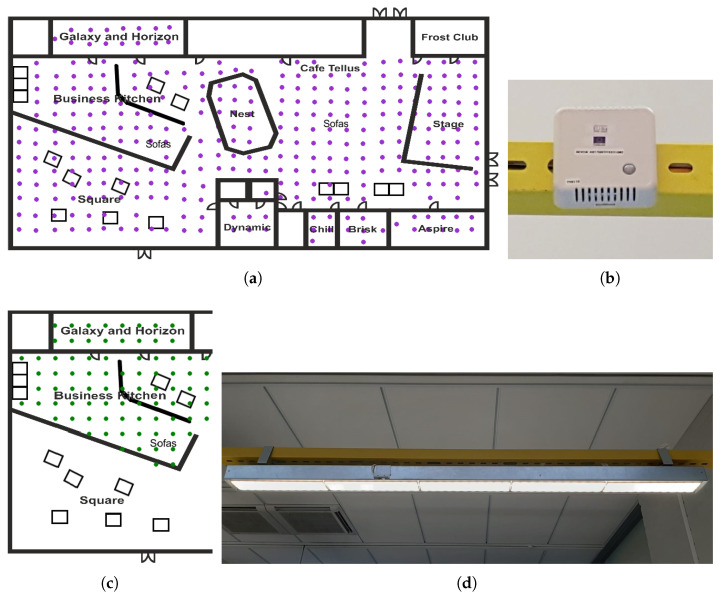
Sensor nodes and luminaires deployed in Tellus Innovation Arena. (**a**) The location information of deployed sensor nodes; (**b**) The sensor node; (**c**) The location information of the luminaires; (**d**) The luminaire.

Tellus includes various kinds of capacities, like meeting rooms, open stage area, study, and rest areas, Figure 1. Each Tellus space has its own dedicated functionality. For instance, some rooms are closed spaces that can be booked and used for meetings, like Aspire, Frost Club, Brisk, Chill, Edge, Galaxy, and Horizon, Figure 2. These spaces are equipped with tables, chairs, projectors, and speakers. Other spaces can be booked for larger events up to 80 people and have additional properties. For example, Stage space has an actual small stage, and Business Kitchen has a number of facilities for collaborative work. Square, in turn, does not need to be booked beforehand. It is an open space for students’ team and individual work, and there are also computers available. Nest is a place for relaxation and thinking; therefore, no tables and chairs are placed there but beanbag seats. Café Tellus provides coffee and tea facilities with comfortable seating places. In addition, there are few cubes located in Tellus (indicated with small squares in Figure 2a); these are tiny closed spaces for about four people that can be booked. Cubes have a desk, board, and sometimes even displays (a cube is visible at the right side of Figure 1b).

We use Low Power Wide Area Network (LPWAN) technology to implement IoT use cases. Altogether, 331 LoRa Wide Area Network (LoRaWAN) sensor nodes are deployed into Tellus premises, each measuring temperature, humidity, CO${}_{2}$, motion, and light, see Figure 2. In addition, each sensor node is geo-positioned, providing us capabilities for spatial analysis. Each node is powered by the two 3.6 V AA lithium batteries and attached to the ceiling frames of Tellus premises. These sensor nodes are placed in a grid with approximately two-meter spacing between each other, as can be seen from Figure 2. Frost Club room does not have sensors due to the difficulties of attaching them to the ceiling in this room. Additionally, some staff rooms and infrastructure facilities are not equipped with the sensors.

The deployed sensor nodes send data packets every 15 min to a remote server utilizing a LoRaWAN radio access network technology. Transmitting on the 868 MHz ISM band, a LoRA gateway manufactured by Multitech is used to collect data from all of the nodes. The gateway is connected to an external biconical D100–1000 antenna from Aerial Oy, and it has an antenna gain of 2dBi. The collected sensor data from this gateway are then transferred using MQTT protocol to the ThingWorx commercial cloud platform, which is then queried by python scripts to store the data on the local campus server. This local campus server is equipped with RDBMS PostgreSQL server for data storage, R, Shiny Server, and Django REST Framework. More in-depth technical details regarding deployment setup and analysis are provided elsewhere [62,63]. Nevertheless, the information that we provide here should be sufficient to obtain a basic understanding of the deployed infrastructure.

In addition to the sensor nodes, Business Kitchen, Galaxy, and Horizon are equipped with a total of 84 Greenled Omega 44 W luminaires (Figure 2d) placed in a grid with approximately 2.0 m horizontal and 2.3 m vertical spacing (Figure 2c). The luminaires are controlled by ActiveAhead (https://helvar.com/solutions/activeahead/) control units. Each control unit is equipped with its own motion detector and a light sensor that enable energy savings by turning off the light when the area is not occupied and by adjusting the luminaire’s lighting based on the variation of the natural light during the day. The control units are able to communicate with each other through a low-energy Bluetooth mesh network and they can learn the general routes people tend to walk to pre-emptively light up the luminaires. Furthermore, the luminaires can be used as actuators through an interface to the mesh network provided by the ActiveAhead manufacturer Helvar Oy Ab.

### 3.2. Data Patterns

**Figure 3:**
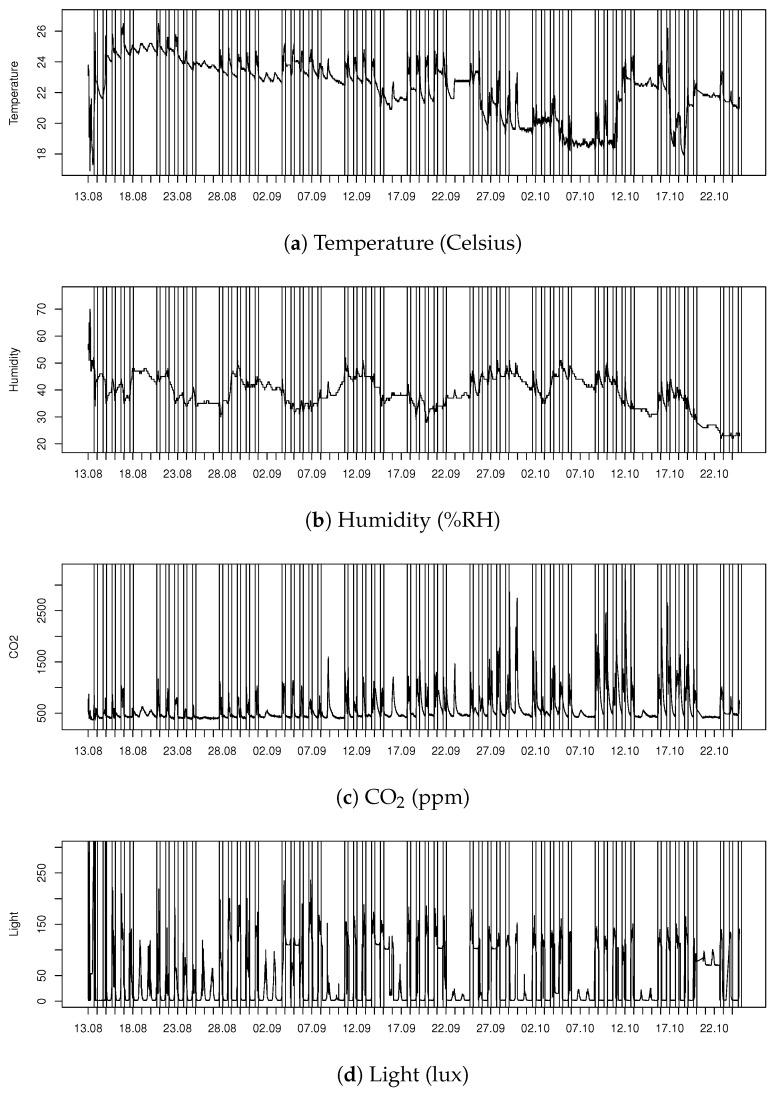
The seasonality in atmosphere measurement time series (work hours have been highlighted).

In this subsection, we describe general patterns of Tellus sensor data. Data in Tellus have clear seasonality, day/night, weekday/weekend; e.g., see Figure 3 that presents different measurements describing indoor conditions collected from one sensor node during 13 August–25 Otober 2017. In order to emphasize the peak time in room usage, the workday hours between 8am and 5pm have been highlighted on the figure.

The temperature time series in Figure 3a has daily peaks and the amplitude is higher during the weekdays. As can be seen, the amplitude significantly increases when the university activities fully start in the middle of September, and the seasonal drop of outside temperature also causes a declining trend in indoor measurements.

The season and outside weather conditions affect not just temperature, but also humidity. In the summertime, the indoor humidity is much higher, and during the wintertime, the indoor humidity can be even uncomfortably low. In Figure 3b, we can see that the short autumn period does not have as significant seasonal trend component in humidity as in temperature in Figure 3a. Still, the effect of the people using the room is visible; the daily peaks appear during workdays and sometimes on Saturdays.

Some of the measured features do have a solid baseline that is mainly affected by the time or the room usage. The carbon dioxide (CO${}_{2}$) is heavily affected by the room users, and the baseline is visible during the nighttime (Figure 3c). Very high values indicate that the ventilation should be increased during the most crowded events, as the CO${}_{2}$ values can increase to a very uncomfortable level. The amount of light has mainly daily variation (Figure 3d); the lights are on when the room is in use. The variation in the amplitude indicates that, sometimes, the room is only partially lit. At daytime, some scattered light can be detected during weekends, although the room is not in use. The measurements also reveal when the lights have been left accidentally on for the night.

Overall, the daily patterns vary in different spaces of Tellus, due to the changing load of the rooms (e.g., some rooms may have booked meetings, others may have bigger events, and, furthermore, there are some open spaces where everyone has access to). Therefore, when finding patterns about the behavior, it is important to treat information within each room and closed area separately. Additionally, different areas, different days or times and seasons have a different natural base level in the signals, and the sensors may have differences in their performance capability. As a result, it is not practical to use fixed levels in the measured signals to indicate general uncomfortability of the space.

### 3.3. Features of Deployed Infrastructure for Data Analysis

One classical use for environment monitoring systems is detecting the events, both occurring commonly, as well as anomalous, i.e., finding patterns that do not conform to expected behavior [29,64]. However, real-world deployments provide a number of challenges for data analysis, as data coming from sensors are erroneous, noisy [31], and could even change statistical properties over time, due to sensor quality or change of the underlying phenomena [37]. In this subsection, we will provide a few comments that we found to be important when dealing with real-world data, as well as highlight unique features of Tellus deployment enabling data quality assessment.

**Figure 4:**
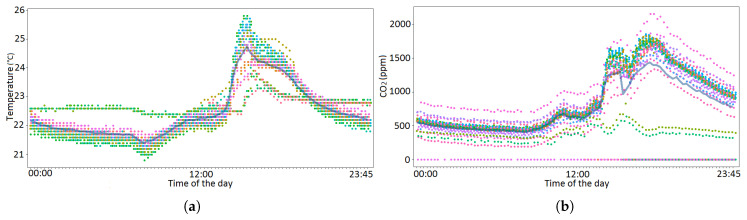
Uncomfortable temperature (**a**) and CO${}_{2}$ (**b**) during one of the events (dots present the raw readings from different sensor nodes in the space).

First, the reliability of the data coming from the sensor should be checked. As an example, consider Figure 4a,b that show the temperature and CO${}_{2}$ readings when one of the spaces in Tellus had a big event. The ambient conditions of the space became quickly unpleasant due to issues with ventilation and a large number of visitors, as we can see from the number of sensor nodes. However, few sensor nodes provide quite different readings when compared to the rest of the space.

**Figure 5:**
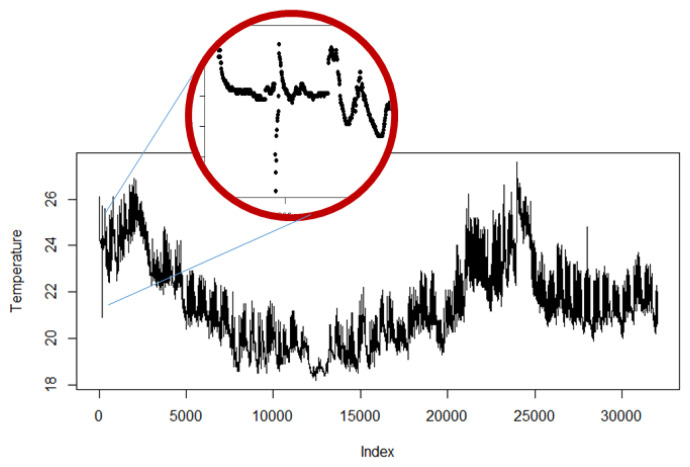
A 1.5 year-long temperature signal containing a couple of abnormal peaks.

The outliers should be detected and removed with automated methods that are feasible for all sensors, variables, and measuring times. Commonly used methods, including gaussian or box-plot based methods, assume the stationarity in the signals, which may not be reasonable for dynamic environments. Large deviations from typical levels are quite easy to remove, but peaks within the plausible area may be trickier. Manually, this work is quite trivial, as humans easily capture sensor malfunctions, but automated cleaning might require several techniques. For example, Figure 5 presents a temperature signal that contains measurements from June 2017 to December 2018. This signal contains at least two abnormal peaks, one at the beginning of the measurement period and the other close to the end. The range of the normal variation in the signal is larger than the amplitude of either of the peaks and, actually, they consist of several consecutive abnormal measurements. Related work also suggests a number of methods [29,55,64], e.g., to use wavelets and neural networks for signal de-noising and anomaly prediction in non-stationary signals [65].

**Figure 6:**
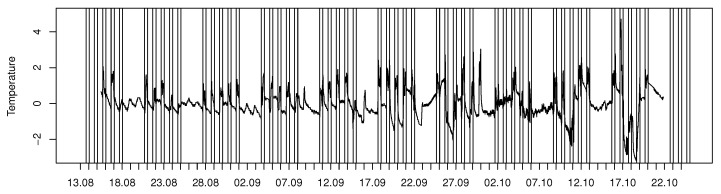
The temperature time series with removed seasonal change trend.

To monitor a large number of sensors automatically, a short history of the signal can be used to determine the typical individual performance for each sensor and measured variable. First, the trend caused by the seasonal change should be removed, if, e.g., the aim was to find the room’s abnormal event. The long-term change can be captured with average filtering and removed from the signal, which enables the daily changes to be monitored. In Figure 6, a trend in temperature time series (Figure 3a) was removed by using a week-long window for averaging. Consequently, daily changes become more visible. Without the trend, the increase in the peak amplitude towards the end of the visualized period becomes clearer, and when combined with the CO${}_{2}$ information (Figure 3c), we can see that the events with a large number of participants affect both temperature and CO${}_{2}$ simultaneously.

The combination of different data sources is useful when detecting an event in the room. For example, it is possible to identify the unintended lighting (Figure 3d) by combining the light measurement with the temperature and CO${}_{2}$ information. If the lights were left accidentally on, the levels of temperature and CO${}_{2}$ should be low, which indicates an empty room.

Tellus IoT deployment provides a number of unique opportunities to assess the quality of retrieved data. First, Tellus sensor nodes are quite densely deployed; this arrangement suggests possibilities to use the neighbor sensor nodes as a reference to estimate the reading of the faulty ones. Second, knowing the event location could suggest only using the sensor nodes of that particular place, to retrieve more accurate readings. Finally, sensor nodes measure different properties of the environment, making it possible to fuse the information for a better understanding of the overall situation.

## 4. Tellus: Insights and Experiments

The collected data provide us numerous opportunities for analysis. For instance, we can learn how Tellus spaces are used and what kind of patterns could be found. In addition, we are able to experiment with data visualization and space interaction. In this section, we report several studies that we have conducted.

### 4.1. Tellus Efficiency

Under efficiency study, we will explore booking system efficiency for Tellus rooms, as well as how lighting is used during working day hours.

The University of Oulu has a room booking system, where a meeting room can be reserved by specifying the room, date, and time interval for the meeting. We conducted a study on booking system efficiency to analyze how the booked rooms are actually used in Tellus. Here, we determine two cases of inefficient use of the booking system. The first case is when the booking was actually made, but no one showed up in the room. The second case of inefficient use is when the actual meeting lasted for much less time than originally booked.

For this study, we analyze the data from Tellus rooms and spaces that are available for booking, namely Aspire, Brisk, Chill, Business Kitchen, and Stage (see Figure 2). Among these, Aspire, Chill, and Brisk are ordinary meeting rooms, available for everyone to book. Business Kitchen and Stage require specific bookings through Tellus staff, and they are more open areas that are booked for large events, like workshops or seminars. Horizon and Galaxy are excluded from analysis, since there is an adjustable wall between the spaces, enabling having a combined room for meetings. In addition, these rooms are booked by Tellus personnel and it is difficult to automatically identify whether reservations made mean combined spaces with the current data, which could lead to misleading interpretation of results.

**Figure 7:**
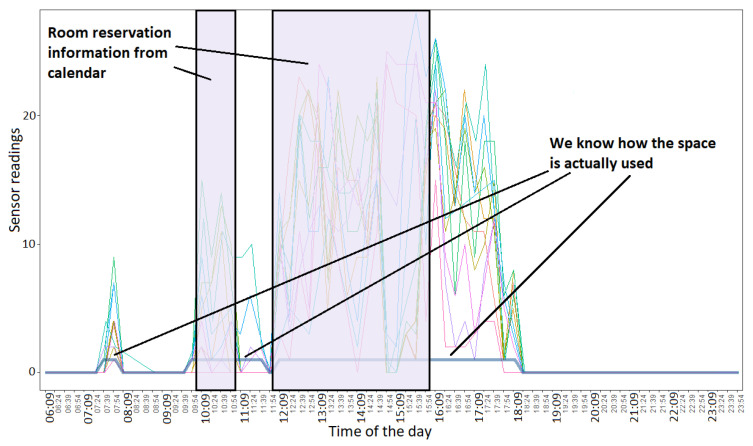
Visualization of the motion and calendar data in particular day in Aspire room.

**Figure 8:**
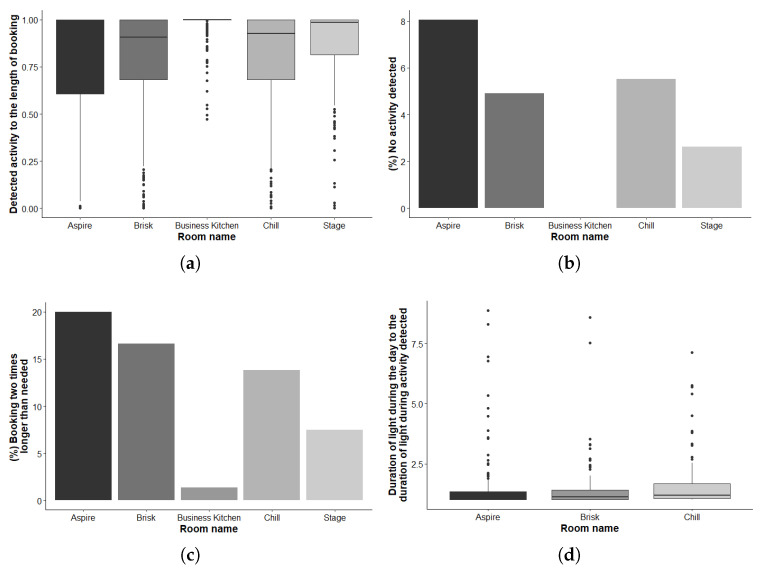
Study results: (**a**) summary statistics for ratio of the time of detected activity to the length of booking; (**b**) percent of cases when no activity was detected (no show) or activity was less than 10 minutes; (**c**) percent of cases when the room was booked for two times longer than needed; and (**d**) summary statistics for ratio of duration of light “on” during the day to the duration of light “on” during activity detected.

For booking system efficiency analysis, we utilize booking calendar data and motion data from the sensors. The time period for the calendar data in this exploratory study is 26 June 2017–17 June 2018. Calendar data were first cleaned from irrelevant booking requests; further, it was aligned with the data for which we did have sensor measurements. To see whether the meeting was actually held, we analyzed motion data with a moving average method with the threshold (Figure 7). Therefore, we could mark the activity occurrences and compare these to the meeting time reserved through the booking system. Figure 8 presents the results. The results presented in this section are supported by Wilcoxon rank-sum tests with a 0.05 significance level.

As can be seen from Figure 8a–c, Brisk and Chill execute similar behavior (W = 111040, *p*-value = 0.8848), when Aspire shows slightly worse results (Aspire/Brisk: W = 65921, *p*-value = 0.0246). As expected, Stage and Business Kitchen demonstrate better efficiency in terms of attending booked meetings compared to other groups. This can be explained by the fact that these spaces are reserved mainly for bigger events which are usually better planned.

To explore the lighting efficiency, we consider the ratio of the time period when the lights in a room are “on” during the day compared to the time period when the lights in a room are “on” during activity detected. Hence, in the ideal situation, this ratio should be close to 1: the lights are “on” during meetings and “off” otherwise. Stage and Business Kitchen spaces are not considered here, since they are open spaces. In this case, activity period determination is the same as with the booking efficiency case. Figure 8d demonstrates the results for the meeting rooms considered. As can be seen, all of the rooms demonstrate excessive use of lighting. Here, Chill demonstrates slightly worse results when compared to the rest of rooms (Chill/Brisk: W = 15934, *p*-value = 0.0032). It seems easier to keep the lighting “on” when someone suspects that another meeting will take place afterwards. We consider such behavior to be a common case for such public spaces where people may also feel to be guests in the space and not to dare to turn off the light.

### 4.2. Space Usage Patterns

**Figure 9:**
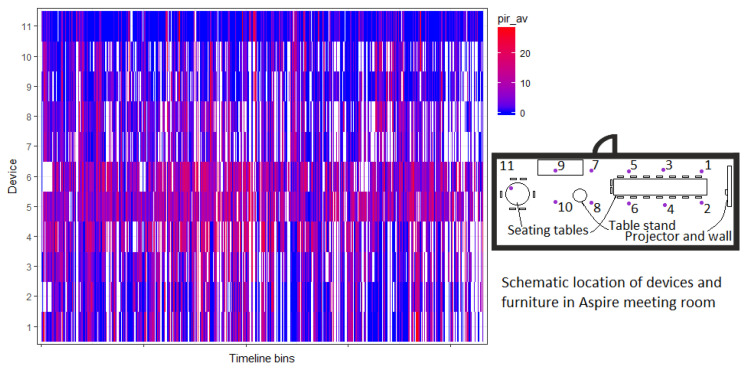
Average pir readings during meetings ordered over 15-minutes time bins.

The collected sensor data allow us to study also interaction patterns in Tellus, for instance, where visitors tend to locate themselves in the rooms. In this case, we analyze the data from the same time interval as in the previous section. Consider, e.g., Aspire room, see Figure 2. This room contains three tables, one is of rectangular size, close to the projector wall, second is a small table stand, and the third one is a round-shape table, further away from the projector. If we plot motion sensor measurements during meetings from all of the sensor nodes in the room, we can see that the ones located above the rectangular table provide more motion readings (see Figure 9) as compared to the round table (compare readings from devices 1–6 and 11). This may provide a logical explanation: the rectangular table is more convenient to work with the laptop, it provides more space to fit people, and it is also closer to the often used projector. An increased number of readings at the table places closer to the door (readings from devices 4–6) may result from capturing the passage of visitors to their desired places. Alternatively, this could indicate that it could be more convenient to settle closer to the entrance, especially if the group of people is small. Either way, based on analyzed data, potentials of the small and cozy round table were unexplored.

**Figure 10:**
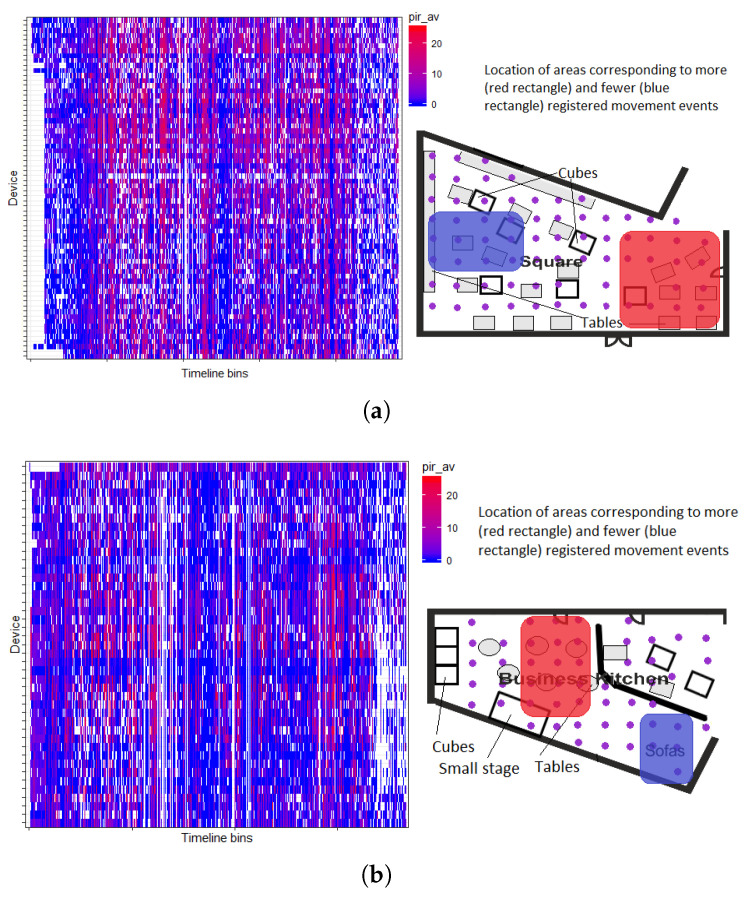
Average pir readings in Square (**a**) and Business Kitchen (**b**) areas over ordered 15-min time bins.

Similarly, we can observe areas generating a higher amount of movement readings in other open places of Tellus, like Square (area for students’ self-studies) and Business Kitchen (place for larger events), see Figure 10. Logically, areas that fall under the pathways, as well as closer to the doors of other rooms, generate more movements. Blue areas (the ones with slightly less registered movements) may provide location preferences (e.g., study atmosphere in Square, Figure 10a) or even perhaps a number of clues, e.g., these areas could be reserved for particular services during some activities happening in the space, since they are slightly less used, Figure 10b.

### 4.3. State Visualization

**Figure 11:**
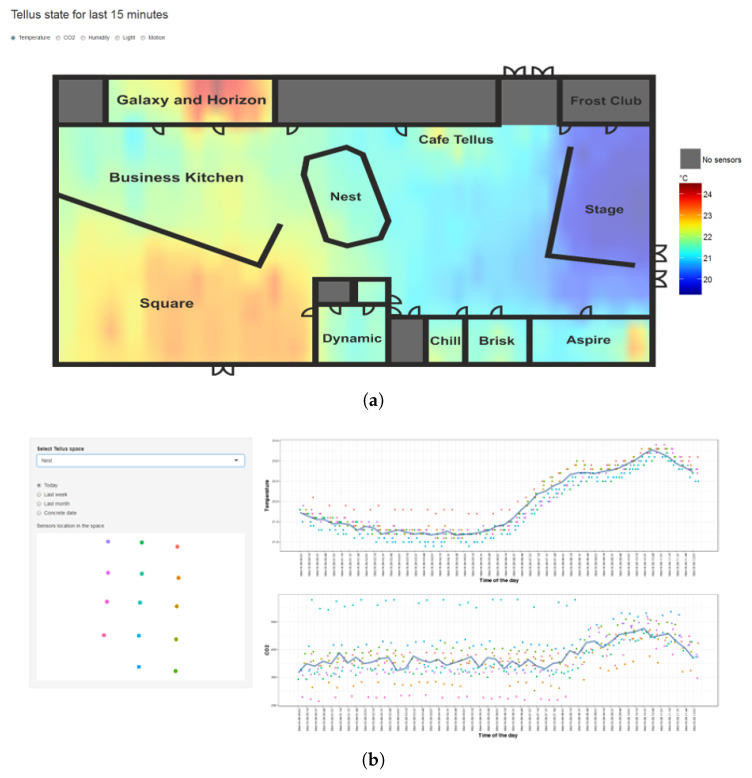
Visualization of Tellus space: (**a**) Heatmap visualization of current state (Temperature parameter selected); and (**b**) basic graphs for the selected space and date.

We have developed two mechanisms to visualize the data to the users in Tellus: dashboard- and AR-based. First, we have provided a basic Web-based dashboard showing the current state of the environment as a heatmap, Figure 11a. Dashboard-based visualization, often accessible via Web interface, appears to be utilized the most in related work, as dashboards provide richer capabilities for analysis [66,67]. In addition, dashboards also allow for the integration of various notification mechanisms [67]. In the dashboard demonstrated with Figure 11, the user is able to select between different properties measured and see the current state of the environment. Inverse Distance Weight (IWD) interpolation is applied to sensed data to generate the heatmap. In addition, the user is able to see the basic graphs for a particular location from the sensors for a certain day, last week, and month (Figure 11b). Such a view can provide a basic awareness of the environment and its patterns. All of the data processing and visualizing scripts are implemented in R with Shiny. This dashboard was publicly presented on the screens located in Galaxy and Horizon spaces and received positive comments from the visitors, since it was easy to interpret the visuals and obtain a quick glimpse of the state in the overall space. However, a color schema for the dashboard, as well as dynamic allocation of the color to the value, should be considered more carefully, since it may provide the wrong impression from a quick view. For instance, the coldest place could be just very few degrees less than the warmest, but if its color presentation differs drastically, it may potentially give an impression of a big temperature difference.

**Figure 12:**
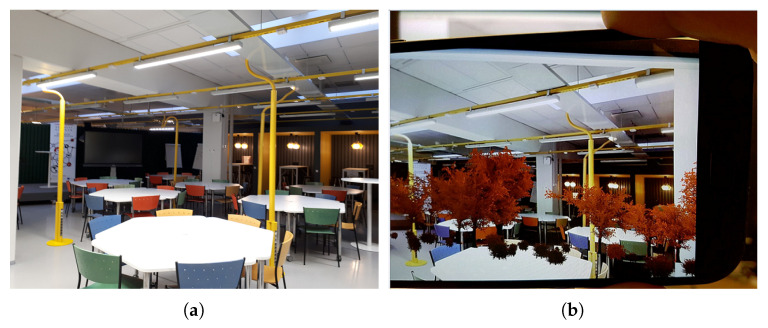
View in Business Kitchen space, (**a**) original view, (**b**) view enhanced with AR objects able to change the color based on the state of the environment.

In addition to the basic dashboard, we have developed AR-based visualization with two prototypes. AR-based solutions promise richer user experience and they are used in various settings, like navigation, tourism, and education [68,69]. The first prototype that we developed demonstrates live sensor measurements when the phone camera is pointing to the particular sensor [63]. The goal of such visualization was to help the maintenance personnel to locate the faulty sensor. User study results demonstrated that for the maintenance purposes, augmenting the view with the actual readings from the sensor could be beneficial to simplify the distinction of the faulty sensors [63]. This is in line with the research of AR potentials in helping to carry out the tasks and interact with IoT objects [69]. The second prototype enriches the actual space with the digital objects that change their properties based on environmental conditions. The use of an abstract object to communicate the information could be challenging. Therefore, we have selected a natural and familiar metaphor (plants) that changes its state (here leaf color) based on environmental conditions, see Figure 12. Other examples of abstractions used for state communication include streamlines entering the room for visualizing indoor air flow [70] or floating spheres showing the CO levels [71]. More research is needed to explore the usefulness of such AR visualization of Tellus state. Similarly to the dashboard case, we may reconsider how to decide the color settings and if they should be personalized. Tellus AR applications are implemented for Android mobile phones with Unity platform and ALVAR for Unity augmented reality plugin (http://virtual.vtt.fi/virtual/proj2/multimedia/alvar/index.html), providing tracking technology.

### 4.4. Space Interaction

**Figure 13:**
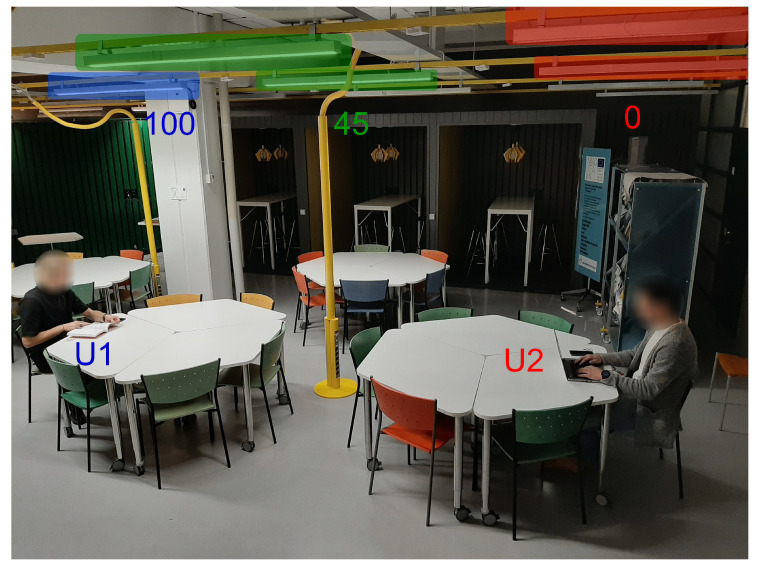
Two users, U1 and U2, use the system. Here, U1 wants to maximize lighting with preference level 100 (maximum light), whereas U2 wants to minimize lighting and has a preference level of 0 (no light). The shared luminaires in between (green) output level 45, since they are a bit closer to U2. The other luminaires in the picture are not in range for these users and since there is no one else in the space, they are turned off.

Smart space interaction has received significant attention from the research community [39,72]. Shared spaces possess certain challenges in adapting the environment, since the preferences of multiple users should be respected. This study explores interaction opportunities with resources of shared smart space, namely with luminaires, see Figure 2c. A prototype aiming to provide lighting control for collaborative spaces was developed for Business Kitchen as part of a Master’s thesis for a newly started Smart Campus initiative at the University of Oulu. This prototype allows users to adjust the lighting settings of the nearby luminaires by using a personal mobile phone. A number of approaches exist to support multiple users in the space and solve possible conflicts, as discussed already in Section 2.1. This prototype uses a weight-based approach, i.e., it treats lighting as a shared resource, and the closest person’s preference has the largest weight to the luminaires outputs in conflict situations (when multiple nearby users control the shared luminaire). When there are no other people nearby using the system, the complete lighting control is given to a single person. Figure 13 shows an example of luminaires’ light intensity control for two users U1 and U2. Both of the users U1 and U2 share two luminaires (indicated with green color in Figure 13); therefore, their value is set to consider the preferences of both users.

**Figure 14:**
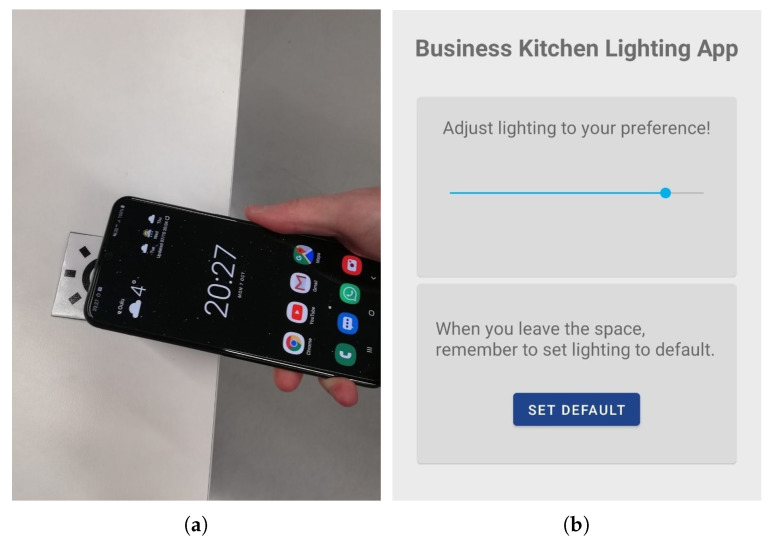
Lighting control prototype: (**a**) Mobile phone touches NFC tag placed on the table; and (**b**) user interface to set the lighting preferences.

The system uses a touch-based interface to initiate the lighting control. That is, the intent to adjust lighting is done by touching NFC tags found at Business Kitchen desks with a smartphone equipped with NFC reader, see Figure 14a. The NFC trigger opens up an Android application, prompts the user to join the Business Kitchen Wi-Fi network if not already joined, and opens up a view to set the user’s personal lighting preference, see Figure 14b. This information is then sent to a server. The server calculates the luminaire outputs based on the users’ preferences and their distances to the luminaires and sends the corresponding control commands to the luminaires. The last person touching an NFC tag receives the full rights to adjust lighting at that place; therefore, the previous occupant’s settings are overridden. When the person occupying the luminaires leaves the range of the Business Kitchen Wi-Fi or gives a system input to leave (“SET DEFAULT” button on Figure 14b), the lighting level is recalculated based on the remaining nearby occupants and is set back to the default level if there are no occupants. If no one is in the space, the ActiveAhead control turns off the luminaire to save energy.

Initial user evaluation was conducted with seven pairs of participants (overall 14 test users) with each pair having tasks to adjust the lighting to a minimum, maximum, medium, and opposite levels in turn. Each participant had control over four luminaires (two with full control and two with shared). NFC tags were positioned in such a way that they were equally distant from the shared luminaires; therefore, the weights for intensity control of shared luminaires were the same for both participants. The study was conducted in the evening time to eliminate the effect of natural lighting from the Business Kitchen roof windows. Additionally, the light intensity of the rest luminaires of the space (not used in the study) was set to level 85, and the lights in nearby cubicles were turned on. The participants were introduced to the system, and then the tasks to perform were given one at a time in a row (a new task was given after the participant had completed the previous task). Finally, the participants were asked to answer the questionnaire.

The results show that nine out of fourteen participants noticed that they were controlling shared luminaires. The rest of the participants did not pay attention to this fact. Two participants perceived the change of light by the other participant disturbing, eight participants were not bothered with it, and four did not have an opinion on the matter. Generally, different lighting levels in the task, surrounding, and background areas do not seem to bother users [73]. However, a variety of factors might affect personal perception, as well as dimensions of the task area. Therefore, more research is needed on the user experience when the system is used by multiple users simultaneously.

Furthermore, seven out of fourteen participants would want to use the system when working in Business Kitchen, whereas three considered the default lighting level to suit their preferences already well enough, and one was afraid to affect other peoples’ lighting. This supports the research that people are trying to avoid conflicts in shared spaces and would rather not use the system at all if there is a risk to negatively affect other participants by adjustments [39].

Half of the participants had not used NFC before; therefore, some practicing was required to get used to this interface. Two users had particular challenges with using NFC technology. However, in the end, only one participant commented that NFC is too cumbersome for this application. Generally, physical browsing technologies (e.g., touching, pointing, scanning) to implement user-to-resource interaction in smart space aim to give a user a feeling of being in control of smart space [72], however, there could be some learning curve to get used to such an interface. For lighting scenarios, being in control also leads to higher satisfaction [39,42]. The participants also gave comments on desired features, like changing light profile preferences (e.g., warm/cold). Generally, the possibility to personalize lighting is positively perceived in related work [74]. The participants also indicated the desire to use a similar control system in other spaces, like home.

### 4.5. Use for Teaching and Development Activities

The wealth of data collected from Tellus suggest possibilities to use this data for education. The environment can be studied from different perspectives, and it offers possibilities for educational activities in several disciplines. So far, three courses with different perspectives and learning outcomes have been offered the opportunity to use the data: Research and Development Project course, Internet of Things course, and Data Mining Project course.

**Research and Development Project course**. In Autumn 2018 and 2019, API to query Tellus data was offered as one potential data source to students for the MSc-level course “Research and Development Project” that aims to teach students to conduct an ICT project professionally.

In the year 2018, the goal of the student project was to explore the available technological opportunities and services of University of Oulu campus and use them to implement Escape Room like prototype. Different research units provided their available technologies to students of this project, and Tellus data were also one potential data source. However, in this case, students did not fully use the provided Tellus data, since the nature of the task (solving puzzle-like situations) required more real-time interaction and sensors in Tellus provide readings every 15 min. However, a system back-end was developed in a way supporting the integration of Tellus data.

**Figure 15:**
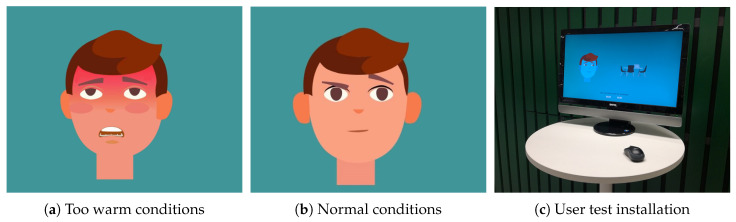
Visualizations from Smart Campus “Provotype” student project work, by courtesy of students.

In the year 2019, another student group was challenged with the development of Smart campus “provotype”. The goal of this project was to create a provocative experience for people at campus. The students decided to visualize the environmental conditions in Tellus with an animated student character, changing his reactions and expressions based on the environmental conditions, and availability of the study spots in Tellus, see Figure 15. The availability of the study places was collected via answering the question of whether the student was able to find the place for study by clicking “Yes” or “No” buttons presented on the same user interface. Depending on the answer, the student character becomes relaxed or more worried, see Figure 15c.

Overall, students found it very useful to work with real-world data for their project work. Knowing environmental conditions is appreciated, especially if the proper applications are developed. Data access API appeared to be convenient; however, students mentioned that availability to query data by location (e.g., by room) would be beneficial. In addition, students pointed out that knowing noise level, as well as a number of people in Tellus, could help in the analysis of student experiences and preferences for utilizing study places.

**Internet of Things course**. Another course that offered Tellus data to students was “Internet of Things”, a first year MSc-level course held in Spring 2019 and 2020. The course introduces basic technologies and novel applications of the Internet of Things. Here, students got a batch of one year collected raw data from Tellus to conduct a data exploration exercise. The goal was to recognize the seasonal variations in the data previously shown in our research studies (especially variations between workdays when space is most crowded), and brainstorm on smart study environment, such as Tellus. Specifically, the following ideas were brought by students from work conducted:

*The smart area could be equipped with auto-adjustable air conditioning providing better conditions for rush hours based on the current situation, not just for average value of the week.* This came from the data observations on CO${}_{2}$ values, showing intensive peaks during the times of high occupancy, especially midday and afternoons of the semester weekdays, defining the periods when air conditioning is the most intensively needed.

*Air humidifiers can be designed and utilized, as part of the Tellus air conditioning solution, to provide optimal healthy working conditions over each season of the year.* This idea came from the analysis of humidity data by students. The significant variation in relative humidity could be mostly explained by significant temperature variation. This is due to the fact that air in the warmer temperatures can hold more moisture than in colder temperatures. Cold winter air (outdoor temperature in the Oulu area can be from −20 to −30 °C during the winter months), even when saturated, contains far less moisture compared to the summer time.

*Seasonal and usage variations could be taken better into account when designing the lighting of the Tellus area.* In optimal conditions, lights could be controlled based on the number of people and their location inside Tellus. “Nightless night”, i.e., full light around the clock around the summer solstice in the Oulu area could also be taken into account by reducing artificial lighting when enough natural light is provided.

Using real data with a research-like exercise statement motivated students to explore and utilize their research skills in practice. The exercise was very open-ended to leave space for students’ own ideas, and they were performed in groups of 3–4 students to gather enough workforce for demanding tasks, including learning new skills, such as large-scale data management, temporal pattern mining, and other ML/analysis tools. Course feedback highlighted that students found the following factors especially useful:

– Working with a real-life, large-scale data set.
– Understanding implementation and operation of smart spaces through concrete work.
– Learning how to develop functional applications for real environments with real end-users.
– Rehearsing innovation and design skills during the group work.
– Possibility to be creative and brainstorm novel innovative ideas.

**Data Mining Project course**. Students conducted more in-depth exploration of Tellus data within the Data Mining Project, a second year MSc level course held in Spring 2020. In this course, the students apply data preprocessing and data mining techniques to real world datasets in 3–4 student groups. The students formulate their research questions and plan the work with the data based on their own interests. In addition, the students needed to manage a research project with all of the steps from data preparation, analysis, and modelling to interpretation and presentation of the results.

In Spring 2020, the students were suggested a Tellus dataset collected from September 2017–November 2018. Therefore, students had the opportunity to tackle the problems associated with real-life data, such as missing observations, errors in measurements, and irregular and non-synchronized sampling frequencies. Students decided to concentrate on healthy atmosphere issues and observed the use of the Tellus spaces and its effect on CO${}_{2}$ concentration. First, the students decided to use hourly averages of the measurements in order to solve the problem of irregular sampling frequencies. They produced 42 to 98 measurements daily, depending on the sensor. They also constructed new variables from the timestamp, such as day of the week and time (night, morning, afternoon, and evening). Because the PIR measurements do not directly tell the number of people in the area, the students experimented with different transformations that might improve the informativeness of the measurement. Additionally, possibilities to aggregate sensor information in order to avoid the use of faulty measurements was considered. Students’ findings include:

*PIR data are the most useful in the more populated areas, like Square.* This is an open area that is the most popular for informal studying in groups, and the students move more frequently than in areas that are dedicated to scheduled events and meetings.

*Finding similarities and differences between usage of the spaces and also between sensors.* Students found out that the data profile of Galaxy differs significantly from other spaces, and Stage and Café, Chill, and Brisk are quite similar pairs but different from other areas, while the rest of the areas formed less significant smaller groups.

*Places and times with highest CO${}_{2}$ concentration.* Areas with highest CO${}_{2}$ concentration, in general, were Brisk, Business Kitchen, and Square.

The students appreciated the possibility to study the environment that they use regularly, and the results can be used for finding healthier work spaces. Possible next steps were also proposed:

– The use of weather data could improve the results of the analysis and the additional information, like speed of users walking or some physiological measurements of the people of the area, could provide valuable information about the health aspects.
– Larger perspective to consider the air quality was proposed as it affects also the building.
– Possibilities to estimate the number of people in the area based on the sensed information.

The course also provided interesting insights for administrative personnel in the university, and the project report was distributed to the coordinator of the Smart campus initiative of the University of Oulu.

**Other activities**. We have also provided a Smart Campus research topic for students in sOULUtions, an interdisciplinary three-day hackathon event held at the University of Oulu in April 2019. This event gathered research and industry customers, as well as students seeking opportunities to demonstrate and learn working-life skills in solving particular challenges. Students were presented problems to work on and they had the opportunity to form groups to focus on one. We challenged students with the question on how to improve study experience and well-being in the campus with the help of smart sensors. The event was actually held in Tellus spaces and sensor visualizations (see Figure 11 and Figure 12) were provided as an example to catalyze creative thinking. The outcome was very promising. A group of students came up with numerous ideas on possible services utilizing sensor data, ranging from improving course management system, finding free parking space, shortest queues in restaurants, and free places for studies. Some of these ideas could be implemented in the future as part of Master theses.

## 5. Challenges and Lessons Learned

Smart campuses combine diverse processes, policies, technologies, and infrastructures. Hence, there are plenty of challenges that could be identified [8]. Here, we list a few challenges and lessons that were learned from employing and using sensor installation into Tellus public open space.

### 5.1. Infrastructure

Infrastructural challenges mostly refer to the technical side of the smart spaces, their installation, management, support, and use.

**Diversity**. Large smart spaces rely on a diverse set of software and hardware stacks, supplied by international and local companies. Some solutions may be provided without proper interfaces, which makes the integration to other services very challenging. This challenge also applies to the data generated by different sensors and services.

All of the 331 sensor nodes at Tellus were from the same vendor and installed at one go. However, since the environment is under continuous development, additional digital solutions are constantly installed. For example, intelligent lights, as described here, were installed later during our research. Deploying new products may lead to interoperability challenges and heavy maintenance costs, and may even limit future use cases and procurement. This is especially true when a diverse set of software and hardware components, e.g., sensors and actuators, are deployed into the same smart space.

Interoperability challenges and middleware solutions for smart spaces have been studied by the research community [15,16,26,27]. At the University of Oulu Smart Campus initiative, we are exploring the architecture and protocols, enabling the integration of existing and new solutions on campus at different levels, like connectivity, semantic, and application-level. For instance, proper data models could allow easier identification of the data sources available and their use for various utility services developed by research units.

**Responsibilities and Support**. This challenge follows directly from the previous one. Who has the responsibility to keep smart space systems and services up and running, especially when we are talking about a smart campus? How to provide the required support? The distributed nature of smart spaces amplifies this challenge. Moreover, how to deal with smart space evolution?

At a university, smart space solutions are usually supported by research staff. This leads to a number of poorly coordinated efforts in creating, advertising, and using of such spaces. It is also challenging to organize proper maintenance support, since research personnel involved in unit projects changes frequently, and skills and accumulated knowledge are lost. Therefore, there is a need for a single point of management of campus digital solutions. A new Smart Campus initiative, started recently at the University of Oulu, aims to improve this situation by introducing structured management plans and detailed descriptions of the planned life cycle of the space (including the development of the space, data collection and usage for research, involvement with the students for educational purposes, and so on).

**Openness of R&D platform**. Smart spaces are excellent research and development platforms, but to which extent can such platforms be open to the public or industry? On the one hand, opening research and development platforms for high-technology industrial experts or the public may bring novel use cases and research projects to universities. On the other hand, could openness lead to a vendor lock-in, where early adopters start perceiving such smart space platforms as a potential market for their products?

Opening a research and development platform requires the development of access principles, protocols, agreements, as well as technical support. The University of Oulu has experience in opening the platforms and infrastructures for education, research, and development, e.g., 5G Test Network+ project [75] and Fab Lab Oulu (university’s fabrication laboratory) [76].

**Security**. Shared smart spaces can be complex distributed systems, which consist of a number of integrated services. Their services and infrastructure must be protected from malicious activities and threats at different levels [77]. Moreover, vital for smart spaces, data must be protected both at rest and in transit. However, authentication policies may complicate finding faults across highly distributed infrastructure during emergency situations [8]. In Tellus, few research groups have only authenticated access to the infrastructure and services. Proper RESTful APIs to fetch the data were developed for students of the "Research and Development Project" course and these APIs were active for the course duration only. Sensor data is not encrypted since there is no personal or sensitive information.

### 5.2. Data Related Aspects

**Privacy**. Privacy, defined generally as "the right to be alone", supports individuals and groups in deciding for themselves when, how, to whom, and what information to share [78]. Privacy has always been a relevant aspect of ambient intelligence and ubiquitous computing research, as there is a trade-off between the collection of personal data and the "smart" capabilities of the services [6]. Transparency and being in control over which information to share are well in line with EU-GDPR [79]. However, transparency and agency could be very difficult to achieve due to the massive scale of open smart spaces [44]. A number of approaches have been suggested to preserve privacy, e.g., obfuscation, anonymization, access control, cryptography, and privacy-preserving data mining [78]. The different methods need to be considered together, since the fusion of various kinds of available data may disclose user sensitive information [80].

In Tellus, only fixed sensor data are currently analyzed. This provides an understanding of the high-level usage of the space. However, with personalized or participatory sensing services development, where analysis of the sensor data from personal devices is included, it might be possible to track users and their preferences. In such a case, a proper consideration of privacy concerns is required, e.g., at the architectural level, similarly to the AnonySense system [81].

**Open data**. Universities possess lots of data that could potentially be useful for improving operational management and utility services to please various stakeholders on campus [7,8]. Of course, the benefits of using the data should be analyzed, as well as proper measures should be implemented to achieve privacy and data protection. Finally, open data should be properly documented and maintained to make it useful.

We gained positive experience in opening Tellus sensor data to students. First, students get knowledge in actual analysis and use of real-world raw data in their projects; therefore, acquiring real-life skills. Second, we obtained an understanding on students’ needs and got fresh ideas for research, services, and teaching development.

**Analysis**. To make decisions, a thorough understanding of the corresponding phenomenon is required [8]. For example, the seasonal changes and weekly dynamics are clearly visible in the Tellus sensor data. Weekly changes show the interesting usage patterns of the space, but the seasonal changes that are caused by the weather could lead to wrong decisions, if not considered in the analysis. Nevertheless, we should accept the fact that some of the information can simply be not captured with the sensing technology available. Therefore, deeper incorporation of qualitative methods and working with stakeholders is required. Second, understanding the causalities involved is challenging. This is where additional context information is of great help. Moreover, real-world long-term deployments challenge data analysis when the environment changes over time. For instance, furniture rearrangements may completely change the pathways that people follow and, therefore, the mobility patterns. Additionally, measurement patterns may change, with furniture rearrangements or when new appliances are introduced to space. Without background information on such spatial rearrangements, one could make faulty conclusions from the data. The availability of several sensors, like in Tellus, helps to capture the changes in a smaller resolution and, therefore, understand the dynamics that are caused by the spatial factors in the space. Additionally, the effect of poorly located sensors or abnormal measurements caused by, e.g., small heat sources, can be recognized and fixed using information from the neighboring sensors. Having only one sensor in the room (which is often the case) limits the analysis and even assessment of the reliability of the measurements received.

Tellus is an open shared space. Over the years, several changes have occurred to this space, as well as available equipment. The largest changes after the installation time occurred in Business Kitchen, Horizon, and Galaxy spaces, where additional equipment was installed and furniture was rearranged. Due to the nature of sensors, we have not noticed abrupt changes, but, nevertheless, we should accept that some misinterpretation of data patterns could happen due to such changes that were not known in advance.

Indeed, drawing conclusions with limited data is a challenge. For instance, when we observed some strange patterns in the data, we always contacted Tellus personnel for clarifications to be sure about the cause. One approach is to fuse different data sources to obtain a fuller picture [82]. For example, in the Booking system efficiency case, we used the calendar booking information. However, this source is not enough if we want, for example, to know the number of people attending the meeting, even though the invited persons are listed in the calendar. For such a scenario, additional data sources are required, for instance, a camera, counting the number of people in the room [83].

All of these observations imply that smart space data analysis is inherently uncertain, and care must be taken to reduce the uncertainty and bias as much as possible. For instance, if uncertainty is too high to make a decision, the system should seek advice from the user [6].

### 5.3. Ethics

Open shared spaces pose a number of questions. For instance, to what extent visitors can be engaged in research studies, and how interventions can affect them [8]? We have already discussed ethical issues in more detail in Section 2.1 and provided some links to the guidelines and initiatives that are available to help embed ethics in the design of the intelligent system. However, it is important to highlight it one more time for future research and the development of smart campus solutions.

Nowadays, to conduct research in shared spaces, universities rely on their internal ethical committees and corresponding policies in terms of what kind of projects are allowed and which measures are needed to ensure proper privacy treatment, check e.g., [83]. According to the assessment by the Ethics Committee of Human Sciences of the University of Oulu conducted at the end of 2017, the use of video cameras in Tellus for research was only allowed with permission retrieved from visitors, i.e., informed consent. Obtaining informed consent from all of the visitors coming to shared space is a challenge requiring either technological or organizational treatment (e.g., use of cameras for closed smaller events only where gathering consent is possible). More research is needed regarding how to ensure that data privacy legislation’s requirements are implemented properly within a smart campus, e.g., a process model was recently suggested for manufacturing industries [84].

### 5.4. User Involvement

Smart spaces can offer many convenient features and services to the user, but they may become easily ignored if they do not sufficiently address the user needs. Therefore, such solutions should follow the human-centered design and implementation approach. Bringing in needs and viewpoints of various stakeholders is needed to design smart space systems. Moreover, users should be able to understand what is happening in the smart space and why, and be able to control it if required [6]. Smart space systems can also utilize users as sensors [85]. The evaluation of personal experience during the stay and interaction with smart space gives valuable information telling how users feel about the environment and whether a smart system helps in accomplishing user tasks. Such information can be explicitly asked from the user or can be automatically inferred from user actions. In any way, such data collection should respect user privacy and be as effortless as possible.

With our educational activities with Tellus data, we were able to obtain some feedback from the students. Particularly, students indicated that it would be nice to also include noise sensing capabilities, e.g., if one wants to have a peaceful place for studies. University of Oulu Smart Campus initiative has initiated discussions with different research units as well, on the research purposes and technical needs, to make the solution as useful as possible.

## 6. Conclusions

This article shared experience in long-term real-world deployment of IoT sensor installation in an open shared place at the university campus. We started from the observation of challenges brought to multi-user open shared smart spaces. We continued with the demonstration of real-world IoT deployment to Tellus Innovation Arena premises of the University of Oulu. Several studies, from data analysis to interaction with the space, were conducted and analyzed in order to demonstrate the possibilities of such installation from both research and educational perspectives. Finally, we have discussed the challenges that we have met and the lessons we have learned. Such an integral overview aims to contribute to the knowledge helping in the design and development of smart campus solutions.

A smart campus initiative that has started at the University of Oulu aims to address the number of issues mentioned in this article, especially from the infrastructure side. Ethical, privacy, and security issues are more challenging and require deeper involvement of different stakeholders. For instance, how can we ensure that smart campus respects data privacy legislation’s requirements? In addition, we are interested in equipping Tellus and other premises of the University with new technologies, like noise sensors, as well as in the development of new utility services. For instance, we plan to explore the possibilities of crowdsourcing solutions to observe ambient conditions of places where we do not have sensor installations. Even though this approach has its own challenges, like the reliability of information [32], we consider it to be viable to obtain an estimate about the state of the environment. Smart space interaction in a shared environment deserves more research. For instance, we may consider equipping Tellus with different technologies that enable physical interaction with resources of smart space, like NFC technology [40]. It is also interesting to explore the opportunities of AR research in different smart space interaction scenarios. Finally, we will also continue investigating the use of smart campus infrastructure for teaching and development activities.
